# Exploring the value of organizational support, engagement, and psychological wellbeing in the volunteer context

**DOI:** 10.3389/fpsyg.2022.915572

**Published:** 2022-09-08

**Authors:** Grace Dekel, Madelyn Geldenhuys, Jemma Harris

**Affiliations:** ^1^Department of Psychological Sciences, Australian College of Applied Professions, Sydney, NSW, Australia; ^2^Department of Psychological Sciences, University of Notre Dame, Sydney, NSW, Australia; ^3^Industrial Psychology and People Management, University of Johannesburg, Johannesburg, South Africa

**Keywords:** not-for-profit organizations, perceived organizational support, psychological wellbeing, volunteer engagement, young adulthood

## Abstract

In Australia, young adults are more likely to experience psychological distress than other age-groups. Accordingly, volunteer work engagement may act as an important tool for supporting psychological wellbeing. The present study relies on the job demands–resources model and self-determination theory to help understand the negative consequences of high work demands and the importance of effective organizational support to enhance positive mental health outcomes. To address research gaps, the current study explores these concepts for the young adulthood cohort in not-for-profit organizations. The study aims to explore the relationship between psychological wellbeing, volunteer work engagement, and perceived organizational support. The study used a quantitative, cross-lagged, longitudinal method for collecting data from two online surveys completed 4 weeks apart. The inclusion criteria of participants were volunteers who worked a minimum of 4 h a month (on average), resided in Australia, and were between 19 and 40 years old (*N* = 202). The main study findings were that perceived organizational support mediated the relationship between psychological wellbeing at time point 1 and volunteer engagement at time point 2. However, perceived organizational support did not mediate volunteer engagement at time point 1 and psychological wellbeing at time point 2. There were no bidirectional effects between volunteer engagement and psychological wellbeing. The findings contributed to the existing literature, suggesting there are overlaps between support mechanisms and motivation between paid and unpaid work. The practical implications for not-for-profit organizations are the importance of providing organizational support for young adult volunteers to improve wellbeing outcomes. Limitations and future study recommendations are presented.

## Introduction

Over the years, philosophers and researchers have asked why does one help another when there is no visible reward? Various studies have found that helping behavior, such as volunteer work and community service increases psychological wellbeing, self-esteem, life satisfaction, and happiness (Lauri and Calleja, 2019). Volunteer work can be a combination of prosocial behavior and altruism and has significant impacts on both the individual and the community (Lay and Hoppomann, 2015). Psychologists argue that people likely help others for either intrinsic or extrinsic purposes (Legault, 2020). Volunteering for career development is an example of extrinsic reward. Alternatively, the positive emotion experienced after responding to a fire as a rural fire service volunteer would be an example of an intrinsic reward. Therefore, volunteer work is multifaceted in the benefits and consequences it can provide an individual. Helping others can be further explained through prosocial behavior which is considered the “social glue” that enables cohesiveness among people of different ages (Lay and Hoppomann, 2015).

Volunteering, altruism, and prosocial behavior can be regarded as antecedents to positive wellbeing, happiness, personal health, and public health (Post, 2014). The current research refers to volunteer work where an individual freely chooses to provide unpaid services to a community organization (Haski-Leventhal et al., 2018). Volunteer work is an important research topic because it may contribute to increases in psychological wellbeing for the individual and social cohesiveness of the community. The local community and Australian government heavily rely on volunteer contributions across many sectors, including health, youth support, and emergency services (Volunteering Australia, 2021). Volunteers have made significant contributions, although in recent years, there has been a decline in the proportion of Australians volunteering. In April 2021, close to one in four people (24%) engaged in volunteer work; however, this also coincided with the COVID-19 pandemic. In comparison, this number was closer to one in three (36%) people in late 2019 (Biddle and Gray, 2021). This may be partly attributed to the reduction in volunteer hours due to COVID-19; however, Australia also saw a 20% decrease in volunteering hours from 2014 to 2019 (Australian Bureau of Statistics, 2020). Therefore, volunteer organizations would benefit from enhancing their understanding of the volunteer experience to identify effective methods to support volunteers’ wellbeing and improve engagement.

There is a range of theories that explores the human experience with volunteer work. The volunteer process model follows a series of stages that explores the volunteer experience (Omoto and Snyder, 2016). First, the antecedent stage includes the individuals’ circumstances and motivation to volunteer. Second, the experiences stage describes the relationship that develops between volunteers and organizations. Finally, the consequences stage represents whether the individuals had positive or negative outcomes from the experience. Moreno-Jiménez and Villodres (2010) research used this model to explore the potential negative outcomes from volunteer work. They found that total time devoted to volunteering (measured in hours per month and number of months in the organization) related to feelings of being worn out and burnt out. Burnout can relate to negative consequences, including cynicism, exhaustion, and lack of professional efficacy. Moreover, when volunteers were motivated by career development, it predicted higher levels of cynicism, whereas when they were motivated by learning new skills, it had the opposite effect with cynicism. The study is limited as it did not identify interventions that could support volunteer wellbeing and retention in the organization. Thus, the volunteer process is important in predicting the mental health outcomes of the volunteer; in addition, the motivation of volunteering appears to be an important factor.

The self-determination theory (SDT; Deci and Ryan, 1980) suggests that one’s behavior is motivated by wanting to satisfy their basic psychological needs for autonomy, relatedness, and competence. Autonomy refers to freely choosing the activity, while relatedness refers to the need for social cohesion, and competence refers to feeling effective in a task. McCarty et al. (2018) conducted a comparative study and found that those who were forced to volunteer devoted significantly less time than those who freely chose to. The theory predicts that those who are intrinsically motivated to volunteer are more likely to have a personally enriching experience than those who are extrinsically motivated. The theory is limited as it cannot be generalized to all cultures. Previous studies in non-Western cultures have not supported the SDT to the same extent as in Western cultures (Ghose and Kassam, 2014).

The functional approach identifies specific types of motivations and examines how this affects the volunteers’ psychological outcomes (Clary et al., 1998). This approach suggests that it is important for the psychological outcome of the volunteer experience to match the individual’s motivation to volunteer. For example, if an individual’s motivation to volunteer is to meet new people and later develop good friendships, they will likely be satisfied by their volunteer experience. This was supported by Finkelstein (2008) who found that volunteers reported greater satisfaction the more their experiences matched their reasons for helping. In addition, those that had greater fulfillment also increased the amount of time they devoted to volunteering work. There was a weak relationship between career goals and volunteer satisfaction; this may be reflective of the age-group which had a mean age of 65 years. Therefore, the study and theory appear to be limited in predominately showing effects for an older age-group, which may not be reflective of the broader volunteer population. Thus, the current study aims to further investigate the generalizability of these findings in the young adulthood contexts.

Various studies have investigated the benefits of volunteering across different life stages. Van Willigen (2000) found a positive relationship between the number of hours spent volunteering and self-reported levels of life satisfaction in older volunteers (aged 60 years and over). Alternatively, for the younger volunteers (aged under 60 years), there was a negative relationship, which suggests that different types of motivation and engagement exist between these two age-groups. However, despite these differences, the current literature tends to focus on the volunteer experience of older populations, rather than more comparative samples. One age-group that would benefit significantly from a more focused investigation is younger adults.

Erikson’s (1994) theory of psychosocial development suggests that the young adulthood group (19- to 40-year-olds) is a formative life stage, and it is important for the individual to develop their sense of identity. Schwartz et al. (2021) identified that during young adulthood, there is a greater risk of engaging in personally and socially destructive behavior, which may negatively impact integration into full adulthood. According to a recent Australian (2017–2018) survey, 15% of people aged 18–24 years experienced high or very high psychological distress (Australian Institute of Health and Welfare, 2021). Therefore, there is a greater need to create effective strategies to reduce levels of psychological distress in this age-group. The current study aims to better understand the volunteer experience of the young adulthood cohort. Future research needs to encompass a range of measures that capture the motivational and behavioral elements, such as self-reported measures of volunteer engagement, psychological wellbeing, and perceived organizational support.

In previous research, volunteer work has predominately been measured by asking participants to recall how many hours they have volunteered (Son and Wilson, 2012). This method could reveal recall and social desirability bias for individuals who may want to show they volunteer more than they do or cannot accurately recall how often they volunteered. In addition, the number of hours spent volunteering can be a limited measure of the overall volunteer experience, and it does not provide a clear and effective indication of how volunteer work can be related to psychological states of wellbeing.

To address this limitation, the current research considers volunteer work engagement as an alternative psychometric tool (Vecina et al., 2012). Work engagement is a positive affective-motivational state characterized by vigor, dedication, and absorption, which is commonly investigated in organizational psychology (Schaufeli, 2011). Vigor refers to high levels of willingness to invest effort, dedication refers to strong involvement in work, and absorption reflects a pleasant state of total immersion in work. The Utrecht Work Engagement Scale (Schaufeli et al., 2006) when applied in the volunteer context may improve the understanding of the quality of the volunteer experience over simply stating the amount of volunteer work. The current research aims to further validate this measure in the volunteer context and explore whether the engagement can be measured over a short period (i.e., 4 weeks).

Previous studies have used longitudinal data to explore the relationship between psychological wellbeing and volunteer work overtime. The findings of Thoits and Hewitt (2001) revealed a bidirectional relationship between psychological wellbeing and the number of hours spent volunteering. Volunteers with higher psychological wellbeing volunteered more frequently and those who volunteered more frequently were more likely to experience higher psychological wellbeing. Their study refers to the personal wellbeing model (Haski-Leventhal and Bargal, 2008), which highlights the importance of physical and mental health resources to increase the likelihood of community involvement. Volunteer work requires an investment of personal resources to the extent that those volunteers who score higher on self-reported measures of psychological wellbeing may be more likely to volunteer and may also see an increase in psychological wellbeing as an outcome of the volunteer experience.

The longitudinal data of Thoits and Hewitt (2001) were collected across two time points over 3 years. The use of this methodology, to the extent that it is bidirectional, improves the understanding of the relationship between volunteer work and wellbeing. There is no generally appropriate time lag for all relationships. In the present study, a time lag of 4 weeks was employed, which was guided by theoretical and applied considerations. At a theoretical level, previous research indicates that psychological wellbeing can change in as little as 1 month (Podsakoff et al., 2012), as well as the effect of support and job demands on the employee (Cieslak et al., 2007). At an applied level, volunteering might be used as a much-needed mental health intervention for younger adults. There are limited studies that explore the effects of wellbeing in the volunteer context over shorter periods, such as 4 weeks. Therefore, the current study time lag of 4 weeks is guided by these theoretical and practical considerations.

The job demands–resources (JD-R) model provides a comprehensive assessment of the health and motivational indicators of work-related wellbeing, and how these are a function of the work environment (Bakker and Demerouti, 2007). In both paid and unpaid work, an overload of physical and emotional work demands can relate to negative consequences, such as burnout. Burnout is characterized by exhaustion and physical and psychological health problems. In addition, a lack of supportive resources may decrease engagement and motivation at work (Hakanen et al., 2008). Job resources, such as fairness, support, and job autonomy may protect against stress and burnout and increase organizational commitment (Boyd et al., 2011). Cox et al. (2010) established the JD-R model in HIV/AIDS volunteers; the findings suggest that volunteers may experience burnout when job demands are too great. Therefore, further strategies should be developed to address the imbalance of individual resources and job demands in the volunteering context. An example of how organizational support can be beneficial is represented in humanitarian not-for-profit workers.

Aldamman et al. (2019) conducted research on humanitarian volunteers who experienced increased anxiety, depression, and burnout. They investigated whether psychological stress measured by perceived helplessness and perceived self-efficacy was mediated by perceived organizational support and mental health outcomes. The results identified that perceived organizational support was positively associated with mental wellbeing and negatively associated with adverse mental health such that organizational support was a key determinant of the mental health of humanitarian volunteers. Perceived organizational support represented the level to which the volunteers believed their organization respected their wellbeing and valued their contributions. The study was limited by the cross-sectional design, which limits further investigation of the direction of the relationships. For instance, beyond levels of organizational support, humanitarian volunteers experience adverse mental health due to the nature of their work. Consequently, without a bidirectional analysis, it is difficult to draw concrete conclusions.

The current study will extend upon existing research by using a longitudinal design with a bidirectional analysis and a sample of Australian young adult volunteers. The current research has two main objectives: first, it investigates the bidirectional relationship between psychological wellbeing and volunteer engagement (Thoits and Hewitt, 2001), and second, it explore whether organizational support indirectly affects psychological wellbeing and volunteer engagement. The present research addresses gaps including limited research exploring these variables in volunteers from the young adulthood life stage. In addition, volunteer work has predominately collected data on how many hours and how long an individual volunteers, and consequently, there are limited psychometric tools that have been designed to measure volunteer work. The current study attempts to address this by evaluating and replicating work engagement in the volunteer context. In addition, consistent with the JD-R model, the current study argues that organizational support has a significant role in supporting volunteers’ psychological wellbeing and engagement. Therefore, the current research seeks to extend upon existing literature by emphasizing the importance of organizational support in volunteer organizations. The first aim of the present research is to show a bidirectional effect between psychological wellbeing and volunteer engagement over a period of 4 weeks. The second aim is to show that organizational support has a mediating effect between psychological wellbeing and volunteer engagement in the 4 weeks.

**FIGURE 1:**
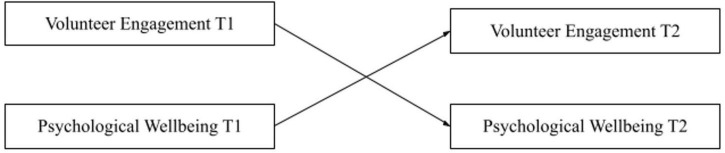
Bidirectional effects model of volunteer engagement and psychological wellbeing. Single headed arrows represent regression, unbroken lines represent direct effects.

**FIGURE 2:**
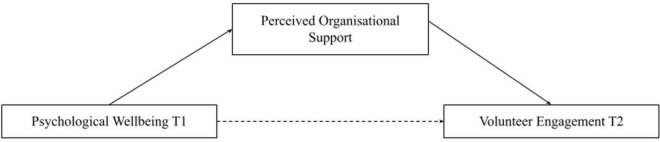
Mediation model of perceived organizational support mediating psychological wellbeing Tl and volunteer engagement T2. Single headed arrows represent regression, unbroken lines represent direct effects, broken lines represent indirect effects.

**FIGURE 3:**
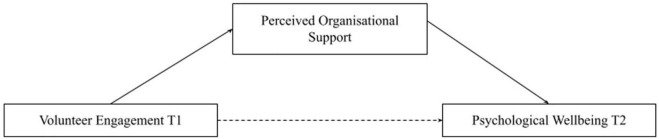
Mediation model of perceived organizational support mediating volunteer engagement Tl and psychological wellbeing T2. Single headed arrows represent regression, unbroken lines represent direct effects, broken lines represent indirect effects.

Hypothesis 1 is that volunteer engagement at time point 1 (T1) has a significant positive direct effect on psychological wellbeing at time point 2 (T2). Hypothesis 2 is that psychological wellbeing at T1 has a significant positive direct effect on volunteer engagement at T2 (see Figure 1). Hypothesis 3 is that psychological wellbeing at T1 and volunteer engagement at T2 will have an indirect effect through perceived organizational support (see Figure 2). Hypothesis 4 is that volunteer engagement at T1 and wellbeing at T2 will have an indirect effect through perceived organizational support (see Figure 3).

## Materials and methods

### Participants

Participants were a convenience sample from the public made up of active volunteers who volunteered a minimum of 4 h a month (on average). The inclusion criteria were individuals aged between 19 and 40 years (*M* = 27.7 years, *SD* = 5.2) residing in Australia. The exclusion criteria were those who did not meet the criteria and were not proficient in English. The sample comprised 138 men (68.3%) and 64 women (31.7%). Further general demographic information is presented in Table 1. Individuals who did not consent or who did not complete both surveys (*n* = 244) were excluded. In total, two surveys were completed by the participants in 4 weeks. This was managed through the Qualtrics survey platform, whereby participants who consented to participate in the second survey were automatically sent an email 4 weeks later with a new survey link. A total of 524 volunteers participated in survey 1, of which 446 provided consent to receive the link for survey 2. Of those 446 participants, 252 completed survey 2. After cleaning the data and matching the participants’ responses from survey 1 and survey 2, 202 participants completed both surveys and consented to their data being used. This led to 404 numbers of occasions.

**TABLE 1:** General demographic information of participants.

Baseline characteristic	Participant demographics	
	*n*	%
Work Status		
Full-time	147	72.80
Full-time, part-time	2	1.00
Part-time	42	20.60
Casual	3	1.50
Contractor, part-time	1	0.50
Contractor	3	1.50
Marital status		
De-facto	2	1.00
Divorced	6	2.90
Married	93	46.00
Never married	100	49.50
Separated	1	0.50
Area of residence		
City	95	47.00
Suburban	85	42.50
Rural	19	9.40
Regional	1	0.50
Level of education		
Never attended school, year 12 or equivalent	1	0.50
Year 11 or equivalent	14	6.90
Year 12 or equivalent	54	26.70
Year 12 or equivalent, certificate III/IV	4	2.00
Year 12 or equivalent, certificate I/II	3	1.50
Year 12 or equivalent, certificate I/II, bachelor’s degree	1	0.50
Certificate I/II	14	6.90
Certificate III/IV	26	12.70
Graduate diploma	11	5.40
Advanced diploma	21	10.30
Bachelor’s degree	19	9.40
Postgraduate degree	34	16.70
Religious status		
Buddhism	25	12.30
Christianity	43	21.40
Catholicism	73	36.00
Hinduism	12	5.90
Islam	26	12.80
Judaism	8	3.90
No religion	14	6.90
Other		
Significant personal life events		
Yes	32	16.80
No	158	83.20

The participants had been volunteering an average of two and a half years (*M* = 30.79 months, *SD* = 14.41) and volunteered on average 4.14 h per month (*SD* = 2.90) between January 2021 and July 2021 when there were minimal COVID restrictions. In total, 34% of participants (*n* = 67) identified as having reduction in volunteer hours due to COVID-19, while 66% (*n* = 130) said they did not. From July 2021, the participants indicated they volunteered an average of 2.20 h per month (*SD* = 1.17). For the second survey (4 weeks later), the participants volunteered 14.17 h in the month (*SD* = 2.03), with an average of 1.75 organization (*SD* = 1.15). Table 2 shows details on how participants were impacted by COVID-19, along with their volunteering status and type of involvement.

**TABLE 2:** Descriptive statistics about participants volunteer involvement and COVID-19 impacts.

Baseline characteristic	Survey one		Survey two	
	
	*n*	*%*	*N*	*%*
Volunteer status	199	98.50	201	99.50
Volunteer involvement				
Freely chosen	159	79.50	189	93.60
Forced to volunteer	2	1.00	0	0.00
Work requirements	35	17.50	10	5.00
University requirements	3	1.50	3	1.50
Episodic volunteer (once off/irregularly)	1	0.50		
COVID-19 impacts				
Self-isolation	76	37.60	9	2.50
Government lockdowns	28	13.90	5	2.50
No impact	95	47.00	96	47.50
Fully vaccinated and able to resume work			74	36.60

### Procedure

This research received approval from the Australian College of Applied Psychology (ACAP) Human Research Ethics Committee (approval number 757200921; see Supplementary Appendix B). Participant recruitment involved online advertisements posted on volunteer-related social media pages (Facebook; see Supplementary Appendix C) and the university recruitment platform SONA for first-year students in psychological sciences. Participants were informed the study involved completing two online surveys investigating the relationship between volunteer engagement, psychological wellbeing, and organizational support (see Supplementary Appendix D). The first survey took approximately 30 min to complete. Those who consented to participate in the second survey were sent the new survey link *via* email 4 weeks after completion. Survey 2 took approximately 20 min to complete (see Supplementary Appendix A).

The participants were informed that they would remain anonymous, and submitting the survey was an indication of their consent to participate (see Supplementary Appendix F). The participants could withdraw by closing the web browser at any point during the survey; however, after submitting responses, withdrawal was no longer possible as names were not tied to responses. In recognition of their time, for each survey in which they participated, the participants were eligible to win one-of-three $40 e-gift vouchers through a voluntary raffle draw, and student participants from ACAP were eligible to receive one credit point. To support participants if they experienced distress, they were provided with a free service sheet (see Supplementary Appendix E). The debriefing page contained further study information and directed participants to the study’s Facebook page for results (see Supplementary Appendix G). Collected data were downloaded from Qualtrics and analyzed using SPSS v. 27 and PROCESS v. 4 for SPSS.

### Statistical analysis

A linear regression analysis was used to test the bidirectional effects of H1 and H2, using the SPSS data program. To test indirect effects for H3 and H4, a mediational regression analysis was performed using the Hayes PROCESS approach with bias-accelerated bootstrapping (Preacher and Hayes, 2004). Jose (2016) recommends the Hayes PROCESS approach in testing longitudinal mediation as it provides a valid estimate of the indirect effects through bootstrapping. The bootstrapping method used 5,000 bootstrap samples of randomly selected observations from the data set that was drawn with replacements (Lockwood and MacKinnon, 1998). The results from the bootstrap sample were then used to create an estimate and confidence interval for each model path. A confidence interval that did not contain 0 indicated a significant model path (Preacher and Hayes, 2004). We generated an estimate and confidence interval for the path from the predictor variable to the mediator (a path), the path from the mediator to the outcome (the *b* path), the overall mediated path (the a*b path), and finally, the direct effect of the predictor on the outcome after controlling for the mediator (the *c*′ path). We chose this mediation procedure as traditional mediation tests have low power (MacKinnon et al., 2002; Shrout and Bolger, 2002). Internal consistency of each questionnaire was conducted using Cronbach’s alpha coefficient of α = 0.70 as acceptable (Cohen, 1988). For the analysis, bivariate associations among variables were examined using Pearson’s correlation, and correlations were interpreted according to Cohen’s (1988) conventions.

### Measures

The participants completed two similar online surveys (Supplementary Appendix A) which collected eight demographic variables, including gender, age, work status, area of residence, marital status, religious status, and level of education. Volunteer impacts also collected related statistics, including volunteer status, volunteer involvement, and COVID-19 impacts. In addition, the participants completed assessments of psychological wellbeing, life satisfaction, happiness, volunteer satisfaction, organizational satisfaction, volunteer engagement, and perceived organizational support (not included in survey 2).

#### COVID-19

To measure COVID-19 impacts, the participants were asked whether they had experienced a reduction in hours volunteering with a “yes” or “no” response option. The question was “has there been a reduction in the level of volunteer work due to COVID-19?”. If they had a “yes” response, they were also asked to respond to an open-ended statement, “please provide a short sentence description of how your volunteer work has changed due to COVID-19.”

#### Psychological well-being

An eight-item Psychological Well-Being Scale (PWB; Diener et al., 2009) was used to assess the individuals’ level of subjective wellbeing. Items were rated using a seven-point Likert scale from 1 = *strongly agree* to 7 = *strongly disagree*. A sample item is “I lead a purposeful and meaningful life.” Following Diener et al. (2009), items were summed to yield a total, with higher scores indicating higher levels of overall positive functioning. Internal consistency for psychological wellbeing in the current study was strong (α = 0.78).

#### Volunteer engagement

A shortened nine-item Utrecht Work Engagement Scale (UWES; Cronbach’s α = 0.92; Schaufeli et al., 2006) was adopted for the volunteering sample. The scale has been validated in a sample of volunteers, where the terminology “work” was made more specific to “voluntary work” (Vecina et al., 2012). Items were rated using a seven-point Likert scale from 0 = *never* to 6 = *always*. A sample item is “I get carried away when I am volunteering.” Following Schaufeli et al. (2006), each item was summed, where higher scores reflected greater vigor, dedication, and absorption when executing their voluntary work. In the present study, the reliability coefficient for volunteer engagement was strong (α = 0.89).

#### Perceived organizational support

A shortened eight-item perceived organizational support (POS; Eisenberger et al., 1986) scale was used to assess the globality of n employees’ or workers’ perception of how they are being supported. The scale was adapted to the volunteer context, where “the organization” was replaced with “the volunteer organization.” The shortened adopted measure was used in a volunteer sample by Aldamman et al. (2019), with Cronbach’s α = 0.83. Items were measured on a seven-point Likert scale from 0 = *strongly disagree* to 6 *strongly agree*. A sample item is “The volunteer organization values my contribution to its well-being.” Higher scores indicated a more positive perception of organizational support. The internal reliability of the scale was sub-par, α = 0.61. To adjust for the low internal consistency, a new variable was created with five items, and the items removed were based on whether the corrected item total correlation was *r* < 0.30 (Hajjar, 2018). When removing items 4, 6, and 8, Cronbach’s alpha improved, and the final internal consistency was strong (α = 0.72).

## Results

### Preliminary analysis

The final sample included 202 participants who completed both surveys and therefore 404 data entries, sufficient for testing a longitudinal model with a complex cross-lagged design (Fields, 2013; Newsom, 2015). This is a sufficient number as some attrition rate is acceptable due to the longitudinal nature of the design (Hedeker et al., 1999). An initial process of listwise deletion in SPSS was conducted on survey 2, and 52 participants were removed from the data because they did not consent, their USERID did not match, or they did not complete the survey. Missing value analysis using Little’s missing completely at random test was conducted on the final sample, revealing data were missing at random, χ^2^(96) = 87.51, *p* = 0.720 (Fields, 2013).

### Normality and assumptions

Table 3 displays descriptive statistics that include means, standard deviations, standard skew, kurtosis, outliers, and Shapiro–Wilk scores. Data from psychological wellbeing at T2 and volunteer engagement at T1 were normally distributed as Shapiro–Wilk tests were non-significant. However, psychological wellbeing at T1, volunteer engagement at T2, and perceived organizational support were not normally distributed as they yielded significant Shapiro–Wilk tests. Kline (2011) suggests that skewness between −3 and 3, and kurtosis between −10 and 10 were appropriate when the sample was more than 200. This recommendation was appropriate, given that the sample consisted of 202 participants who completed two surveys which sum to 404 data points. According to Kline (2011), psychological wellbeing at T1, psychological wellbeing at T2, and volunteer engagement at T1 did not violate normality. However, volunteer engagement at T2 and perceived organizational support were positively skewed.

**TABLE 3:** Summary table of normality assessment for variables.

Variables	*M*	*SD*	Modality	Skewness	Kurtosis	Outliers	Shapiro–Wilk
Psychological wellbeing T1	43.5	5.1	Unimodal	−4.25	8.10	8 with | *z*| > 1.96 (4.1%). 2 with | *z*| > 2.58 (1%). 3 with | *z*| > 3.29 (1.6%).	*W* (160) = 0.93***
Psychological wellbeing T2	42.5	4.7	Unimodal	−0.46	−1.18		*W* (160) = 0.99
Volunteer engagement T1	36.6	6.4	Unimodal	−0.50	1.49		*W* (160) = 0.99
Volunteer engagement T2	28.1	7.4	Unimodal	5.44	1.45	13 with | *z*| > 1.96 (6.7%). 3 with | *z*| > 2.58 (1.5%).	*W* (160) = 0.94***
Perceived organizational support	13.6	4.5	Unimodal	5.08	1.09	4 with | *z*| > 1.96 (2.1%). 4 with | *z*| > 2.58 (2.1%).	*W* (160) = 0.93***

Researchers conducted further analysis to assess outliers in the variables. Psychological wellbeing at T1, volunteer engagement at T2, and perceived organizational support had univariate outliers (see Table 3). Winsorizing was implemented to deal with these outliers. Any data value above the 95th percentile was replaced by the value of the 95th percentile, and any value lower than the fifth percentile was replaced by the value of the 5th percentile (Ghosh and Vogt, 2012). After winsorization, psychological wellbeing at T1 improved the Shapiro–Wilk score, perceived organizational support, and volunteer engagement at T2 yielded improvement in skewness and kurtosis, although not in Shapiro–Wilk scores. Table 4 represents the new means and standard deviations. Homogeneity of variance was met for both predictors, multicollinearity was met, and no multivariate outliers were identified in the dependent variables. There were no other issues of mathematical assumptions (see Supplementary Appendix H).

**TABLE 4:** Means, standard deviations, and normality statistics for winsorized scores.

Variables	*M*	*SD*	Skewness	Kurtosis	Shapiro–Wilk
Psychological wellbeing T1	43.4	4.2	0.34	−0.38	*W* (175) = 0.98*
Volunteer engagement T2	28.2	6.9	4.08	0.41	*W* (175) = 0.93***
Perceived organizational support	13.5	4.4	3.70	−0.52	*W* (175) = 0.92***

N = 202, the outliers of original scores were trimmed by changing scores over 95th and 5th percentiles to the respective scores.

*p < 0.05, ***p < 0.001.

### Bivariate correlations

There was an overall trend of weak–moderate correlations (see Table 5). There was a significant weak negative correlation between volunteer engagement at T1 and psychological wellbeing at T2. We found a large positive significant relationship between volunteer engagement T1 and psychological wellbeing T1. Psychological wellbeing at T2 and perceived organizational support have a large significant positive relationship. No correlation was found between volunteer engagement at T2 and perceived organizational support, psychological wellbeing at T2 and volunteer engagement at T2, and perceived organizational support and volunteer engagement at T1.

**TABLE 5:** Pearson’s correlations for measures.

Variables	1	2	3	4
(1) Psychological wellbeing T1	–			
(2) Psychological wellbeing T2	0.08	–		
(3) Volunteer engagement T1	0.41**	−0.20**	–	
(4) Volunteer engagement T2	0.10	0.08	−0.17*	
(5) Perceived organisational support	0.39**	0.34**	−0.05	0.16**

*p < 0.05, **p < 0.01.

### Bidirectional effects

Hypothesis 1 was tested using linear regression analysis. The results showed that volunteer engagement at T1 had a negative significant effect on psychological wellbeing at T2. Although a significant direct effect was found, the hypothesis was not met since this relationship is negative. Volunteer engagement at T1 had a negative significant relationship on psychological wellbeing T2, *F*(1,186) = 7.81, *p* < 0.01, *R*^2^ = 0.04, adjusted *R*^2^ = 0.035. The regression coefficient (β = −0.15, 95% CI [−0.26, −0.05]) indicated that an increase in volunteer engagement at T1 corresponded, on average, to a decrease in psychological wellbeing at T2 of 0.15 points. Hypothesis 2 stated that psychological wellbeing at T1 would be positively significantly predicted by volunteer engagement at T2, to test whether a linear regression analysis was used. The hypothesis was not met, psychological wellbeing at T1 was not significantly predicted by volunteer engagement at T2, *R*^2^ = 0.01, *F*(1,182) = 1.65, *p* = 0.201, 95% CI [−0.09, 0.39].

### Mediation analysis

**FIGURE 4:**
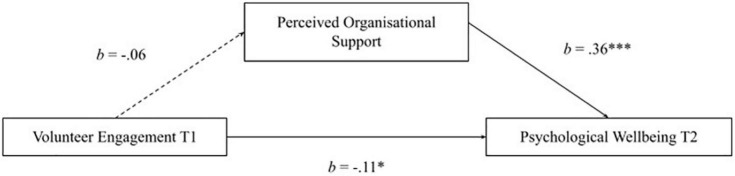
No mediation model of volunteer engagement Tl and psychological wellbeing T2 through perceived organizational support. No mediation in the model, *n* = 202. Unbroken lines are direct significant pathways between variables; broken lines represent indirect pathways between variables. Unstandardized effects are shown and represented by *b* values. *p < 0.05, ^***^p < 0.001.

Hypothesis 3 stated that volunteer engagement at T1 and psychological wellbeing at T2 would be mediated by perceived organizational support. The first model which tested Hypothesis 3 resulted in no mediation (see Figure 4). There was a non-significant relationship between volunteer engagement at T1 and perceived organizational support, *R*^2^ = 0.008. There was a significant positive direct effect between perceived organizational support and psychological wellbeing at T2. In addition, volunteer engagement at T1 and psychological wellbeing at T2 had a significant negative effect when mediated by perceived organizational support, *R*^2^ = 0.14^***^ (weak effect). Contrary to prediction, the indirect effect of perceived organizational support on volunteer engagement at T1 and psychological wellbeing at T2 was not significant, *b* = −0.02, BCa 95% CI [−0.08, 0.02].

**FIGURE 5:**
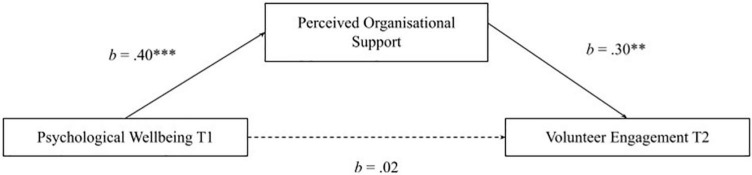
Full mediation of psychological wellbeing Tl and volunteer engagement T2 through perceived organizational support. Final model of mediation effects measuring young adults’ psychological wellbeing time point 1 (Tl) and volunteer engagement time point 2 (T2) through perceived organizational support, *n* = 202. Unbroken lines are direct significant pathways between variables; broken lines represent indirect pathways between variables. Unstandardized effects are shown and represented by *b* values. ^**^p < 0.01, ^***^p < 0.001.

Hypothesis 4 stated that psychological wellbeing at T1 and volunteer engagement at T2 would be mediated by perceived organizational support. The second model which tested Hypothesis 4 yielded a full mediation effect (see Figure 5). There was a significant positive direct effect of psychological wellbeing at T1 on perceived organizational support, *R*^2^ = 0.15^***^ (weak effect). In addition, a significant positive direct effect of organizational support on volunteer engagement T2, *R*^2^ = 0.04* (weak effect). The pathway (*c*′) between psychological wellbeing at T1 and volunteer engagement at T2 mediated by perceived organizational support was not significant. Following the prediction, the indirect effect of perceived organizational support on psychological wellbeing at T1 and volunteer engagement at T2 was significant, *b* = 0.12, BCa 95% CI [0.009, 0.249].

### Additional analyses

A multiple linear regression analysis was conducted to investigate perceived organizational support as a predictor of psychological wellbeing at T1 and T2, and volunteer engagement at T1 and T2. Table 6 shows the regression coefficients of these analyses. Perceived organizational support significantly positively predicted psychological wellbeing at T2 (medium effect size) and psychological wellbeing at T1 (medium effect size). Volunteer engagement at T1 had a negative significant effect on perceived organizational support (weak effect size), and volunteer engagement at T2 was not statistically significant.

**TABLE 6:** Multiple linear regression coefficients of perceived organizational support on wellbeing and engagement.

Variable	*B*	β	*SE*
Constant	−13.98		4.02
Psychological wellbeing T1	0.42***	0.40	0.08
Volunteer engagement T1	−0.11*	−0.16	0.05
Psychological wellbeing T2	0.26***	0.29	0.06
Volunteer engagement T2	0.08	0.13	0.04
*R* ^2^	0.31***		
Adj. *R*^2^	0.29		

N = 202. We examined the impact of perceived organizational support on psychological wellbeing and volunteer engagement across time point 1 and time point 2.

*p < 0.05, **p < 0.01, ***p < 0.001.

An additional multiple linear regression was run to identify the impact of whether a reduction of volunteer hours due to COVID-19-predicted change in psychological wellbeing at T1 and T2, as well as volunteer engagement T1 and T2. The model was significant and indicated the four predictors explained 14% of the variance [*R*^2^ = 0.14, *F*(4,163) = 6.43, *p* < 0.001]. COVID-19 significantly predicted a decrease in volunteer engagement at T1 (β = −0.24, *p* < 0.05) and a significant increase in psychological wellbeing at T2 (β = 0.24, *p* < 0.05). COVID-19 did not significantly predict psychological wellbeing at T1 (β = 0.13, *p* = 0.104) and volunteer engagement T2 (β = −0.02, *p* = 0.760).

## Discussion

The objectives of this research were to explore whether volunteer work had a significant personal benefit for young adults. Specifically, perceived organizational support indirectly affected the relationship between volunteer work engagement at time point 1 (T1) and psychological wellbeing at time point 2 (T2). We found no evidence to support a bidirectional relationship between volunteer work engagement and psychological wellbeing. There was a negative relationship found between volunteer work engagement at T1 and psychological wellbeing at T2. Furthermore, we found no significant relationship between psychological wellbeing at T1 and volunteer work engagement at T2. Regarding indirect effects, psychological wellbeing at T1 did not have an indirect effect on volunteer work engagement at T2 through perceived organizational support; however, volunteer work engagement at T1 had an indirect effect on psychological wellbeing at T2 through perceived organizational support. This study addressed research gaps because previous studies had not used these variables in the model, did not use a time lag of 4 weeks, or the young adulthood sample.

### Bidirectional effects: Hypothesis 1 and hypothesis 2

Although the relationship between volunteer work engagement at T1 and psychological wellbeing at T2 was significant, no bidirectional relationship between volunteer work engagement and psychological wellbeing was found. Therefore, the results of this study do not support previous studies that explored the bidirectional effects between volunteer work and psychological wellbeing over longer periods of 3 and 10 years (Thoits and Hewitt, 2001; Son and Wilson, 2012). The direct effect between volunteer work engagement at T1 and psychological wellbeing at T2 was negative. Therefore, suggesting a decrease in psychological wellbeing over time, while no significant effect was found between psychological wellbeing at T1 and volunteer work engagement at T2. This result supports hypothesis 3 that the third variable of perceived organizational support may indirectly affect the relationship between psychological wellbeing at T1 and volunteer work engagement at T2.

Volunteer work engagement at T1 and volunteer work engagement at T2 were significantly negatively correlated, which shows a negative relationship with engagement over time. This suggests that there is likely variability in how volunteers engage and how their wellbeing changes across different time points. There may be contributing organizational factors, such as unexpected changes in the volunteer work environment that limited the participants’ ability to develop high levels of engagement. In addition, there was no correlation between psychological wellbeing at T1 and T2. Previous research has found psychological wellbeing to be variable overtime, although it is relatively stable over shorter periods (Cieslak et al., 2007). In addition, participants may have been in a generally good state of wellbeing, and it may take more than one variable time point to perceive a severe change in wellbeing.

Previous studies applied the JD-R model (Bakker and Demerouti, 2007) to understand the positive and negative outcomes for employees and volunteers. When there are greater demands than resources, this may negatively impact psychological wellbeing and volunteer work engagement. In the current study, the participants may have been exposed to greater demands in the form of personal stressors, which may be reflected by hours spent volunteering. In the current survey, additional analyses suggested that COVID-19 significantly impacted volunteer work engagement at T1 and psychological wellbeing at T2. The participants were asked how their volunteer hours changed throughout the year. They reported volunteering an average of 4 h per month between January 2021 and July 2021 before COVID-19 restrictions, which reduced to 2 h per month during July 2021, and then increased to 14 h at T2. This could suggest that the increase in hours volunteers worked increased the demand and workload for the individual, negatively impacting their engagement and wellbeing. In addition, there were greater stressors associated with this period due to COVID-19, including lockdowns, higher risk of infection, and change of in-person volunteer activities to online forums. Volunteer work engagement is a state of absorption, dedication, and vigor in which individuals feel fulfilled by their task (Schaufeli, 2011). When reaching a high level of work engagement, individuals may often feel energized after the experience, developing a sense of fulfillment and greater wellbeing. The discrepancy in findings may be attributed to the 19- to 40-year-old sample, which is likely busier than that outside of this age-group. Their lifestyle may include a combination of work and personal commitments, including full-time work, a young family, and higher education studies. These personal and social factors may contribute to the challenges of immersing oneself in volunteer work.

### Indirect effects: Hypothesis 3 and hypothesis 4

Hypothesis 3 was not supported as there was no indirect effect of wellbeing and engagement through perceived organizational support. The unexpected results may be due to a weak correlation between engagement at T1 and T2 in the current study, which likely resulted in limited interaction with the organization. During the period of data collection (July – October 2021), there were higher rates of COVID cases, alongside local government lockdowns. Thus, volunteers likely spent less time with organizations in general. In the model, perceived organizational support significantly predicted psychological wellbeing at T2, which suggests that those who observed that the organization valued their contributions were more likely to present with an increase in psychological wellbeing at T2. Moreover, through further analyses, perceived organizational support had a positive relationship with psychological wellbeing at T1 and psychological wellbeing at T2, although a weak negative relationship with volunteer work engagement at T1. These findings suggest that perceived organizational support may have a greater effect on wellbeing than engagement.

Hypothesis 4 was supported as perceived organizational support had a significant indirect effect on psychological wellbeing at T1 and volunteer work engagement at T2. These findings are in line with previous studies as they suggest that perceived organizational support contributes to volunteer mental health through its effect on wellbeing and engagement (Aldamman et al., 2019; Pahlevan Sharif et al., 2021). Windsor et al. (2008) suggested that volunteers in the young adulthood life stage (younger than 60 years) do not derive the same psychological benefits as older adult volunteers (over 60 years; Windsor et al., 2008). The present study contrasts these results and indicates that a presence of perceived organizational support may mediate the relationship between wellbeing and engagement for younger volunteers. Similarly, Clary and Snyder (1999) suggest those in young adulthood are driven to volunteer for extrinsic purposes, such as developing skills and making connections for their careers. The current study findings did not support this as most participants (79.50% in the first survey and 93.60% in the second survey) indicated they were freely choosing to volunteer, in contrast to being extrinsically motivated by work requirements. In survey 1, 17.50% of the participants indicated they were volunteering for work requirements, but this reduced in the second survey to 10%. The change in volunteer involvement may be a consequence of organizational support such that when volunteers have well-established resources, it may encourage them to continue volunteering of their own volition.

### Implications of the findings

The contributions of the present research are the previously unexplored variables of work engagement in the volunteer context for the young adulthood cohort tested using the regression and mediational model. Findings from this study produce opportunities for further investigations into the relationship between these variables, across different time lags, and demographics. These findings contribute to both theory and empirical evidence as they provide support for Bakker and Demerouti’s (2007) JD-R model. The current findings encourage a balanced approach where not-for-profit organizations should implement strategies to provide appropriate support resources. In addition, the present study further supported the SDT (Ryan and Deci, 2000), which suggests that when volunteer work is freely chosen and the individual has good relatedness in the form of perceived organizational support, then they may experience competence in the form of engagement.

The practical implications from this research suggest that on an individual level, people may experience a positive relationship between psychological wellbeing and volunteer work engagement with the presence of organizational support. For example, organizational management may proactively provide feedback to volunteers to show them that their work is being appreciated and recognized. In addition, organizations can provide weekly or monthly “check-ins” by assigned mentors where volunteers can express any concerns they may have. There are potential implications for the young adulthood cohort in Australia that encourage volunteer participation. The present findings depict an increase between levels of freely chosen volunteer involvement from time point 1 to time point 2. This suggests that young adults that freely choose to volunteer may derive personal benefits from the experience. Organizations may choose to provide greater resources to support the young adulthood demographics to increase volunteer work engagement and wellbeing. These findings may inform not-for-profit organizations on how to develop better recruitment and volunteer retention strategies for the young adulthood cohort.

The macro-level implications of the study are the Australian ideologies for prosocial and helping behavior in the local and wider community. Not-for-profit organizations would benefit from increased funding to hire high-level professionals, such as organizational psychologists to advise on how to best support their volunteers. There may be smaller volunteer organizations that cannot learn and create avenues that will ensure professional development, organizational support, and subsequently volunteer retention. Thus, the present study contributed to the volunteer and not-for-profit organizational research field from a multitude of levels that can lead to further research.

### Limitations and future directions

The present study did not use a control group of non-volunteers. Therefore, the study was not able to assess whether the results were significantly different from the natural changes of engagement, wellbeing, and support over time. Hence, the current study does not imply causation, and the findings were correlational. The current model can be used in future research to assess whether similar findings are present in a non-volunteer sample working in for-profit organizations. In addition, there are limitations in generalizing Western findings to non-western populations. Hence, future research can investigate how the current model fits with young adult samples from different ethnic and cultural backgrounds. There was a negative correlation of volunteer engagement between the two time points, as well as no correlation between psychological wellbeing across time points. These unexpected results represent a limitation of the cross-lagged effects as they do not explain weekly or monthly variations, in comparison to multilevel analyses accounting for lagged effects. Therefore, future research should measure across multiple time points to assess the variability over time of volunteer engagement and psychological wellbeing for the young adulthood cohort.

The study was not able to measure the emotional and cognitive impacts of the COVID-19 pandemic. The current study measured the behavioral impact of COVID-19 through the reduction in hours of volunteer work. However, it is important to acknowledge that the volunteer experience may have been impacted to a greater extent by COVID-19, for example, by necessitating work from home with limited social interaction and the fear of contracting COVID-19. The period of data collection may have contributed to the unexpected findings, and future research should explore the psychological impacts of COVID-19.

The type of volunteer work was not measured in this study. Therefore, the study cannot provide organizations with specific recommendations that reflect the nature of their volunteer work. Future studies should focus on types of volunteers, for instance, comparing the levels of engagement and wellbeing between emergency service volunteers and volunteer telehealth workers. Finally, there is much research required on the impact of volunteer work in young adulthood such that future research should explore the relationship between wellbeing and other psychological states associated with unpaid work. For example, burnout can contribute to the understanding of how to improve volunteer retention and organizational commitment, further enhancing the volunteer experience for this age-group.

## Conclusion

This study was the first to investigate the bidirectional effects between psychological wellbeing and volunteer engagement across a time lag of 4 weeks, and the mediating effect of perceived organizational support of psychological wellbeing and volunteer engagement for a young adulthood sample. The findings highlighted the importance of organizational support in mediating the relationship between psychological wellbeing and volunteer engagement, providing new insights into the volunteer experience of young adults. Future research should replicate the present model and include new variables to assess the psychological impacts of COVID-19, re-testing the variables across multiple time points, and exploring new volunteer-related outcomes in the young adulthood life stage. Nevertheless, the present findings are significant and contribute to a richer understanding of the factors supporting volunteer work in young adulthood. Indeed, it appears that helping others can help oneself, with the appropriate support resources in place.

## Data availability statement

The original contributions presented in this study are included in the article/Supplementary material, further inquiries can be directed to the corresponding author.

## Ethics statement

The studies involving human participants were reviewed and approved by Australian College of Applied Professions Human Research Ethics. The patients/participants provided their written informed consent to participate in this study.

## Author contributions

GD, MG, and JH contributed to the conception and interpretation of the study and results. MG developed the study design. JH assisted with framing the literature review. GD managed the data collection, performed the analysis, and wrote the completed drafts of the manuscript. All authors contributed to manuscript revision, read, and approved the submitted version.
