# Supplementary material for: Exploring the value of organizational support, engagement, and psychological wellbeing in the volunteer context
Source: Front Psychol. 2022 Sep 8;13:915572. doi: 10.3389/fpsyg.2022.915572 (PMC9496652; doi:10.3389/fpsyg.2022.915572)
Supplement: Supplementary file 2 [file Data_Sheet_2.pdf]

Appendix A: Survey Instruments

SECTION 1 – Demographic Questions

Nine demographic questions to complete for survey one at Timepoint 1. Nine demographic questions will be included in survey two at time point 2 with asterisk (*).

1. Age*: How old are you in years?

2. Gender*: How would you describe your gender?

Male	Female	Non-binary	Another term (please specify)

3. Marital Status*:
   - ☐ De Facto
   - ☐ Divorced
   - ☐ Married
   - ☐ Never Married
   - ☐ Separated
   - ☐ Widowed
   - ☐ Decline to answer
4. Work status*: You may select more the one option
   - ☐ Full time
   - ☐ Part time

- ☐ Casual
- ☐ Contractor

5. Location*: Where is your home located?
   - ☐ City area
   - ☐ Suburban area
   - ☐ Rural area
   - ☐ Regional area
6. Education: What is your highest level of completed education? Please select all applicable
   - ☐ Never attended school
   - ☐ Year 11 or equivalent
   - ☐ Year 12 or equivalent
   - ☐ Certificate I/II
   - ☐ Certificate III/IV
   - ☐ Bachelor's degree
   - ☐ Graduate Diploma / Graduate Certificate
   - ☐ Advanced Diploma / Diploma
   - ☐ Postgraduate Degree
7. Physical health*: How would you describe your physical health?
   - ☐ Poor
   - ☐ Fair
   - ☐ Good
   - ☐ Very good
   - ☐ Excellent

8. Religious status: With which religion do you identify?

    - ☐ Buddhism
    - ☐ Christianity
    - ☐ Catholicism
    - ☐ Hinduism
    - ☐ Islam
    - ☐ Judaism
    - ☐ No religion
    - ☐ Decline to answer
    - ☐ Other

9. Personal circumstances*: Have you experienced any significant life events that may impact your ability to volunteer?

    - ☐ Yes

      If yes, please specify how__________

    - ☐ No

SECTION 2 – COVID-19 Information

In Australia 2021, the local government area restrictions and stay-at-home orders may have impacted your ability to participate in regular volunteering. Please answer the following questions with considering to what extent these factors have impacted your experience:

1. Since July 2021, have you been required to adopt any (or all) of the following COVID-19 restrictions:
   - ☐ Self-isolation due to personally contracting COVID
   - ☐ Self-isolation due to being a close contact of someone who has contracted COVID
   - ☐ Self-isolation due to being a casual contact of someone who has contracted COVID
   - ☐ Living or working in a local government area that has stay at home orders
   - ☐ Self-isolation due to being immune-suppressed or having vulnerable family members
   - ☐ Not at all
2. Has there been a reduction in level of volunteer work due to Covid-19?
   - ☐ Yes
   - ☐ No
   - ☐ Not sure
3. If yes, please provide a short sentence description of how your volunteer work has changed due to COVID-19: ____

SECTION 3 – Volunteering Questions

Instructions: volunteer work is an unpaid, freely chosen proactive activity which requires time commitment and effort. Please respond to the following five questions with your voluntary work and not-for-profit organisation in mind:

1. Volunteer status: Do you currently participate in volunteer work?
   - ☐ Yes
   - ☐ No
2. How long have you been volunteering for? Please include months and years (if applicable)

_____Months ________Years

3. Volunteer hours: Between January 2021 – July 2021, on average how many hours do you volunteer **per month** (overall)?
   - ☐ 2 – 4
   - ☐ 4 – 6
   - ☐ 6 – 8
   - ☐ 8 – 12
   - ☐ 12 +
4. Volunteer hours: Between July 2021 – now, on average how many hours do you volunteer **per month** (overall)?
   - ☐ 2 – 4
   - ☐ 4 – 6
   - ☐ 6 – 8
   - ☐ 8 – 12
   - ☐ 12 +

5. How would you categorise your volunteer involvement (please select one option that best represents your circumstances)?
   - ☐ I freely choose to volunteer
   - ☐ I am volunteering due to work requirements
   - ☐ I am volunteering due to TAFE/university requirements
   - ☐ I am an episodic volunteer (once off/irregularly)
   - ☐ Other (please specify) _______
6. Organisation: How many not-for-profit organisations do you volunteer with?
   - ☐ 1
   - ☐ 2
   - ☐ 3
   - ☐ 4+
7. Volunteer satisfaction: how satisfied are you with your volunteer work? In this context, consider whether your personal expectations and needs are being/have been met. If you work for multiple organisations, please consider the location you volunteer at most:
   - ☐ Very dissatisfied
   - ☐ Dissatisfied
   - ☐ Neutral
   - ☐ Satisfied
   - ☐ Very satisfied
8. Volunteer satisfaction: How satisfied are you with your volunteer organisation? In this context, consider whether your personal expectations and needs are being/have been met. If you work for multiple organisations, please consider the location you volunteer at most:

- ☐ Very dissatisfied
- ☐ Dissatisfied
- ☐ Neutral
- ☐ Satisfied
- ☐ Very satisfied

SECTION 4 – Psychological Wellbeing Measures

SECTION 4A – Psychological wellbeing scale

Instructions: Below are 8 statements with which you may agree or disagree. Using the 1–7 scale below, indicate your agreement with each item by indicating that response for each statement

Rating Scale

Strongly agree	Agree	Slightly agree	Neither agree nor disagree	Slightly disagree	Disagree	Strongly disagree
7	6	5	4	3	2	1

**STATEMENTS**	**RATING**
1. I lead a purposeful and meaningful life.	
2. My social relationships are supportive and rewarding	
3. I am engaged and interested in my daily activities	
4. I actively contribute to the happiness and well-being of others	
5. I am competent and capable in the activities that are important to me	
6. I am a good person and live a good life	
7. I am optimistic about my future	
8. People respect me	

SECTION 4B – Satisfaction with Life and Happiness Scale

Instructions: this inventory consists of 5 sentence items. Read each sentence and decide how often the feeling was present over the past few weeks, according to the following graded response scale:

Never	Almost never	Rarely	Sometimes	Often	Very often	Always
0	1	2	3	4	5	6

STATEMENT	RATING
1. In most ways my life is close to my ideals	
2. The conditions of my life are excellent.	
3. I am satisfied with life.	
4. So far, I have gotten the important things I want in life.	
5. If I could live my life over, I would change almost nothing.	

SECTION 4C - Happiness Scale

Instructions: For each of the following statements and/or questions, please circle the point on the scale that you feel is most appropriate in describing you.

STATEMENTS
1. In general, I consider myself
Not a very happy person 1 2 3 4 5 6 7 a very happy person
2. Compared with most of my peers, I consider myself
less happy 1 2 3 4 5 6 7 more happy
3. Some people are generally very happy. They enjoy life regardless of what is going on, getting the most out of everything. To what extent does this characterization describe you?
not at all 1 2 3 4 5 6 7 a great deal
4. Some people are generally not very happy. Although they are not depressed, they never seem as happy as they might be. To what extent does this characterization describe you?
not at all 1 2 3 4 5 6 7 a great deal

SECTION 6 – Volunteer Engagement Scale

Instructions: The following 9 statements are about how you feel when participating in voluntary work. Please read each statement carefully and decide if you ever feel this way about your volunteer organisation. If you have never had this feeling, cross the "0" (zero) in the space after the statement. If you have had this feeling, indicate how often you felt it by crossing the number (from 1 to 6) that best describes how frequently you feel that way.

Never	Almost never	Rarely	Sometimes	Often	Very Often	Always
0	1	2	3	4	5	6

**STATEMENTS**	**RATING**
1. At volunteering, I feel bursting with energy	
2. At my volunteer organisation, I feel strong and vigorous	
3. I am enthusiastic about my voluntary work	
4. My voluntary work inspires me	
5. When I get up in the morning, I feel like going to volunteering	
6. I feel happy when I am volunteering intensely	
7. I am proud of the voluntary work that I do	
8. I am immersed in my voluntary work	
9. I get carried away when I am volunteering	

SECTION 7 – Perceived Organisational Support

Listed below are statements that represent possible opinions that YOU may have about working at your volunteer/not-for-profit organisation. If you volunteer with multiple organisations, please consider where you volunteer most of your time. Please rate the degree of your agreement or disagreement with each statement that best represents your point of view about your volunteer work. Please choose from the following answers:

Strongly agree	Somewhat agree	Agree a little	Neither agree nor disagree	Disagree a little	Somewhat disagree	Strongly disagree
6	5	4	3	2	1	0

STATEMENTS	RATING
1. The volunteer organisation values my contribution to its well-being	
2. The volunteer organisation fails to appreciate any extra effort from me. ®	
3. The volunteer organisation would ignore any complaint from me ®	
4. The volunteer organisation really cares about my well-being	
5. Even if I did the best job possible, the volunteer organisation would fail to notice. ®	
6. The volunteer organisation cares about my general satisfaction at work.	
7. The volunteer organisation shows very little concern for me ®	
8. The volunteer organisation takes pride in my accomplishments at work	

Appendix B: HREC Approval

NOTICE OF OUTCOME

NAVITAS PROFESSIONAL INSTITUTE HUMAN RESEARCH ETHICS COMMITTEE

REVIEW OF RESEARCH APPLICATION

**Research Applicant's Name**	Chen Dekel
**Research Applicant's Email**	256854@my.acap.edu.au
**Research Supervisor's Name**	Madelyn Geldenhuys Jemma Harris
**Research Supervisor's Email**	Madelyn.Geldenhuys@acap.edu.au Jemma.Harris@acap.edu.au

APPROVAL

☒ The NPI HREC has completed its ethical review and approves your proposed human research to proceed as proposed.

Please provide a progress report to hrec@navitas.com by March 20, 2022

Your NPI approval number is 757200921

Your NPI approval expires September 20, 2022

WITHHELD

☐ The NPI HREC has completed its ethical review and is withholding your approval until you submit a revised application and explanatory letter addressed to the:

A. Chair ☐

B. Committee ☐

Please see the next page for the requirements for resubmission and what is required for the submission either to be reviewed by the HREC or by the Chair.

EXEMPT

☐ The NPI HREC has completed its ethical review and exempted your proposed research from ethical review. You are not required to liaise further with the NPI HREC concerning your planned research

REJECTED

☐ The NPI HREC has rejected your application on ethical grounds

Date of feedback from the NPI HREC: September 20, 2021

Dr Fiona Ann Papps
Chair, NPI HREC

PROJECT TITLE:

Does Helping Others Mean Helping Yourself? The Effects of Volunteer Engagement, Wellbeing, and Volunteer Engagement

NOTE: Approvals from sites at which researchers plan to advertise must be forwarded to the NPI HREC for their records.

Any correspondence regarding your project should include project approval number and project title.

The Committee

Appendix C: Facebook Advertisement

Please visit link to access website:

https://www.facebook.com/Does-Helping-Others-Mean-Helping-Yourself-104205358590073

Appendix D: Research Participant Information Statement

**Research Study Title**	Does Helping Others Mean Helping Yourself? The Effects of Volunteer Engagement, Psychological Wellbeing, and Organisational Support
**NPI HREC Approval Number**	TBC
**Researcher's Name**	Chen (Grace) Dekel, Dr Jemma Harris, Dr Madelyn Geldenhuys
**Researcher's Relationship to ACAP**	Student

(1) What is the study about?

This study seeks to explore how volunteer engagement is related to psychological wellbeing, and alternatively how psychological wellbeing relates to volunteer engagement. In addition, to see whether organisational support indirectly relates to volunteer engagement and wellbeing. Previous studies mainly focussed on older adults, and how organisational support relates to paid work and not in the volunteer context. The current study aims to uncover further information including investigating the relationship between psychological wellbeing and volunteer work in the short term (i.e., 4 weeks). Moreover, to focus on the psychological wellbeing of young adults in Australia.

(2) Who is carrying out the study?

Grace Dekel, a student at the Australian College of Applied Psychology (ACAP), is carrying out the research. The research is part of studies toward the Bachelor of Psychological Science (Honours) degree. She is working under the co-supervision of Dr Jemma Harris and Associate Professor Madelyn Geldenhuys in the Discipline of Psychological Sciences at ACAP.

(3) What does the study involve?

If you decide to participate in the study, you will participate in two similar online experiments over the Qualtrics website. The first survey you can complete at any time over the first week of October.

The survey will include questionnaires about general demographics, volunteer details, psychological wellbeing (Psychological wellbeing, life satisfaction, and happiness), volunteer engagement, and perceived organisational support. The second survey will be distributed on the final week of October (four weeks later). This survey will be shorter and have the same questionnaires as mentioned before, except not including perceived organisational support questionnaire, and religious status (demographics).

For the survey you will create a unique identifier code (USERID) that will be used when completing the second survey. You can choose to participate in the second survey at the end of the first survey. To participate an email address will need to be provided, your personal information will not be linked back to your original responses.

All participants can choose to enter a raffle draw with a 1 in 3 chance of winning a $40 egift voucher (per survey). Participants entering through SONA are also eligible for 1 course credit (per survey). Personal identifiers such as student details, and email address are managed by ACAP administration staff and always kept secure.

(4) How long will the study take?

The first survey will take 30 – 40 minutes to complete. The second survey and will take 20 – 30 minutes to complete. Overall, participation in the study will be approximately 1 hour.

(5) Will I incur any costs by participating in this study?

No. There are no costs associated with participating in this study.

(6) Are there any risks attached participating in the study?

In the survey, you will be asked questions about:

- Your identity – sex, gender, age etc.
- Psychological wellbeing – personal wellbeing, life satisfaction, and happiness
- Volunteer experience
- Your experiences with the ongoing COVID-19 pandemic

These questions may cause you distress. Only you can determine what your response might be when you reflect on the questions you will be asked, and therefore only you can determine the level of emotional distress that taking part in this research might pose for you.

Please consider the impact engaging in this research may have. You can choose to exit the survey at any time by closing the browser window. You can skip any questions that you do not feel comfortable to answer. If you do experience distress, a list of free support services will be accessible to you on the survey portal.

(7) Can I tell other people about the study?

You may share with anyone you wish that you participated in the study.

(8) Will I receive the results of the study?

The results of the research will be available after *March 6th, 2022*. Please check the social media page for the research after this date for a release of the results (***https://www.facebook.com/Does-Helping-Others-Mean-Helping-Yourself-104205358590073***). You can also contact the Primary Investigator Dr Jemma Harris jemma.harris@acap.edu.au, or (02) 9964 6379.

(9) Confidentiality and disclosure of information

There is some risk associated with data storage in this study, which will be held securely off-site. All data will be de-identified, and no names or personal identification of participants will be recorded or associated with test results.

All participants are eligible to be included in a raffle draw with a 1 in 3 chance to win a $40 egift voucher. Also, SONA participants receive course credit for participating. To maintain confidentiality for these incentives, personal information is stored separately from participant responses. A new window will open linked to a different Qualtrics survey where you can provide your email address. These responses are not linked to your survey response to maintain anonymity. Data de-identification of participant responses will also ensure that the information you provide will not be linked back to you or your not-for-profit organisation. Data from this research will be retained for at least five years to be able to validate or replicate the research, and to prove ownership of intellectual property.

You cannot be identified from any of your results. We will keep the information you provide confidential. However, the NPI Human Research Ethics Committee (a committee that reviews and approves research studies) may inspect and copy records pertaining to this research. If we write a report about this study, we will do so in such a way that you cannot be identified. Submitting your responses indicates that you have agreed to participate in the study.

(10) Can I withdraw from the study?

Participation in this study is voluntary - you are not under any obligation to consent and your decision to withdraw at any point will in no way affect your relationship with the Australian College of Applied Psychology. You will not incur any penalty from withdrawing whether that be credit points (students), or ability to enter raffle draw.

Please note that completing the online study and submitting the completed questionnaires is an indication of your consent to participate in the study. You can withdraw any time prior to completing the tests or submitting your completed questionnaires by closing your browser window. However, once you have completed the tests or submitted your questionnaires anonymously, your responses cannot be withdrawn. This is because your responses are not attached to you in anyway, so it is not possible to identify your responses.

(11) How will my responses be used?

Your responses will be used to test a model to assess whether there is a bidirectional effect of volunteer engagement and psychological wellbeing. In addition, exploring whether organisational support indirectly effects the bidirectional effect of volunteer engagement and psychological wellbeing. The results of the research will be used to compile a 12,000-word Thesis for completion of my Honours degree. The data may be used in future research to test for replication or further research in the field.

(12) How can I obtain further information?

If you have any questions or concerns, please contact the researchers at the social media page for this research (https://www.facebook.com/Does-Helping-Others-Mean-Helping-Yourself-104205358590073).

(13) What can I do if I have a complaint or concern?

Any concerns or complaints about the conduct of this study should be directed to:

NPI HREC Secretary
Email: hrec@navitas.com

Any complaint will be investigated promptly, and you will be informed of the outcome.

This information sheet is for you to keep.

Appendix E: Consent Statement

Remember that this survey is not a test, and so your responses cannot be "right" or "wrong". Your name or any other identifying information in this survey will not be retained, and the IP address recorded against your survey responses will not be recorded. Therefore, you will not be able to be identified by any of your responses.

Once you have completed the survey, you can choose whether you wish your data to be included. If you choose to allow your data to be used, once you submit your responses to the survey, they cannot be removed from the study.

Remember that even though everything outlined above is done to protect the security of your responses, no computer transmission can be perfectly secure.

You can withdraw from the research at any time by closing your browser window, and your responses will not be recorded or used by the researchers.

I have read and understood the information provided to me. I have had my questions about the research answered to my satisfaction.

I am between 19 and 40 years of age	☐ Yes
I live in Australia	☐ Yes
I am proficient enough in English to answer questions in English	☐ Yes
I have read and understood the information provided to me	☐ Yes
I have no personal relationship with the researchers	☐ Yes
I am a current volunteer at a not-for-profit organisation	☐ Yes
I am volunteer a minimum of 4 hours a month (on average)	☐ Yes

I consent to participate.

Yes (Taken to online survey; insert link)

No (Taken to the end of the survey)

Appendix F: Services Sheet

In the course of this study, information is being collected about issues that may have personal significance for some people. If you are troubled by any topic or issue about which information is being collected and you would like support or counselling, please ensure that you seek external support from a counsellor and/or a social service.

If you are an Australian resident the following free services may be able to help:

Service	Website	Contact
Aboriginal Counselling	https://www.aboriginalcounsellingservices.com.au/contact/	0410539905
Aboriginal Contact Line	https://www.victimsservices.justice.nsw.gov.au/Documents/pt12_atsi-contact-line.pdf	1800 019 123
Beyond Blue	https://online.beyondblue.org.au/email/#/send	1 300 22 4636
Eheadspace	https://www.eheadspace.org.au/	
Kids Helpline	https://kidshelpline.com.au/	1 800 55 1800
Lifeline	https://www.lifeline.org.au/get-help/online-services/crisis-chat	13 11 14
Mental Health Line	https://www.health.nsw.gov.au/mentalhealth/Pages/contact-service.aspx	1 800 011 511
Mission Australia Helpline	https://www.missionaustralia.com.au/	1 300 886 999
Q Life	https://qlife.org.au/	1 800 184 527
1 800 RESPECT	https://www.1800respect.org.au/contact-us/	1 800 737 732

Disclaimer: We do not assume any responsibility for the quality of the services offered by these organizations.

Appendix G: Debriefing Statement

Thank you for participating in this research. I consent to my responses being used in this study.

- ❑ Yes
- ❑ No

Summary of research conducted:

This research aims to explore the relationship between psychological wellbeing and volunteer engagement. In addition, how organisational support may impact the relationship between volunteer engagement and psychological wellbeing. Your responses to my research will help me to add to this current area of interest in organisational psychology and volunteering research.

Who is conducting the study?

This research is being conducted by Grace (Legal name: Chen) Dekel at the Australian College of Applied Psychology, Sydney campus, as part of the fulfilment of the assessment requirements toward the Bachelor of Psychological Science (Honours) degree. It is being supervised by Dr Jemma Harris, who is an academic in the Discipline of Psychological Sciences. It is co-supervised by Associate Professor Madelyn Geldenhuys, who is currently acting as the Discipline Lead for the Sydney campus and teaching into the undergraduate and post graduate qualifications.

What are the next steps?

The research team will collate your responses and summarise them. The research team will analyse the survey responses for patterns in the data and for direct and indirect effects of volunteer engagement, psychological wellbeing, and organisational support in Australian young adults.

How will the survey results be used?

The survey results will be used in the write up of a 12,000-word psychology honours thesis. The findings may be used in conference presentations and future publications

Privacy and confidentiality

You will not be able to be identified from any of the information that you provide in your survey, as de-identified data are used in any publication that results from the research. Any IP number attached to your data will be deleted. Data will be stored securely on a password protected USB to which only members of the research team have access for the duration of the research.

The information that is collected from this project will be numeric. Once entered an SPSS data file (similar to excel spreadsheet), This file will be stored on a password protected USB

that only Grace (Chen) Dekel, and Dr Jemma Harris will have access to. Once the research has concluded and the final report submitted to the NPI HREC, the primary investigator will give the password protected USB to the NPI HREC secretary who will then transfer the responses to a protected drive. The USB will then be destroyed. NPI HREC secretary who will then transfer the responses to a protected drive. The USB will then be destroyed. After a period of five years (as recommended by the NHMRC [2007]), NPI-HREC secretary will then delete the data. Therefore, all records of the research will be destroyed by March 2027.

If you would like to know the results of this research, please contact me or Dr Harris in the Discipline of Psychological Sciences at the Australian College of Applied Psychology at jemma.harris@acap.edu.au, or (02) 9964 6379 after March 6, 2022. The results will also be posted on the Facebook research page (https://www.facebook.com/Does-Helping-Others-Mean-Helping-Yourself-104205358590073).

I would like to remind you that if you experience any distress after participating in this research, you are able to contact any of the services on the list that I provided you at the beginning of the research.

Note that some scholarly journals require making the data from research available to them for the purposes of checking the reliability and accuracy of the findings reported in the journal article. As no identifying information is retained during this research, you will not be able to be identified in this data file.

Appendix H: Multivariate Normality Checking

Normal Distribution of Residuals

The histogram of standardised residuals for volunteer engagement T2 appears to be positively skewed, and psychological wellbeing T2 histogram was found to approximate a normal distribution. The histogram of standardised residuals for psychological wellbeing T2 appears normally distributed, which is supported by the P-P plot which has minimal snaking. The mean residuals for both regression models are zero.

Independent Residuals

Independence of residuals appear to be met because the Durbin-Watson statistic for each of the regression models was within range of 1.5 to 2.5 (volunteer engagement T2 = 2.02, psychological wellbeing T2 = 1.88,), indicating no trend of autocorrelation among residuals of the model (Field, 2013).

Homogeneity of Variance

In a plot of the standardised predicted values against studentized residuals of volunteer engagement T2 there was no discernible pattern observed. This conclusion was further supported by significant Breusch-Pagan statistic (Critical χ^2 (1) = 3.841; volunteer engagement T2 χ^2 (1) = 2.22, p < .05). In addition, no pattern was identified in graph depicting standardised predicted values against studentized residuals of psychological wellbeing T2. Further supported by significant Breusch-Pagan statistic (Critical χ^2 (1) = 3.841; psychological wellbeing T2 χ^2 (1) = 1.414, p < .05). Therefore, homoscedasticity was met for both volunteer engagement T2, and psychological wellbeing T2.
